# The natural history of osteogenesis imperfecta: a systematic review

**DOI:** 10.1016/j.bonr.2026.101927

**Published:** 2026-06-05

**Authors:** Davide Gatti, Samantha Prince, Ogün Sazova, Clive Whitcher, Antoinette Randet, Matthew Carter, Maria Rapoport, Oliver Semler

**Affiliations:** a Department of Rheumatology, University of Verona, Verona, Italy; b Mereo BioPharma Group plc, London, UK; c Wickenstones Ltd, Oxford, Oxfordshire, UK; d University of Cologne, Faculty of Medicine and University Hospital Cologne, Department of Pediatrics, Cologne, Germany

**Keywords:** Osteogenesis imperfecta, Natural history, Bone fragility, Fractures, Disease progression, Clinical manifestations, Chronic conditions

## Abstract

Osteogenesis imperfecta (OI) is a rare, heritable condition characterised by bone fragility, varied manifestations, and phenotypic heterogeneity. Understanding its natural history is essential for anticipating clinical needs. This systematic review collated literature on OI's natural history, focussing on diagnosis, signs, symptoms, and events (SSEs), and mortality.

MEDLINE, Embase, and Embase Conference Abstracts were searched on March 24, 2024. Longitudinal (≥5 years follow-up) and cross-sectional studies which analysed outcomes by age were included, irrespective of interventions, due to treatment variability in OI.

Sixty-six studies were included. Age of diagnosis varied widely; severe OI was typically diagnosed in early childhood, whereas milder types showed greater variability. SSEs manifest across ages. Some SSEs progress rapidly in childhood (e.g. scoliosis and bone deformities), whereas others (e.g. cardiac, ocular, auditory, and joint issues) emerge in adulthood, often earlier and more commonly than in the general population. Severe phenotypes may be associated with earlier onset and greater symptom severity than milder types. Fractures may be most frequent during childhood and adolescence but continue into adulthood, with limb fractures most reported. Aging, pregnancy, and menopause may increase the risk of hip, spine, and femur fractures. Life expectancy appears reduced by an average 9.5 years in men and 7.1 in women versus the general population. Leading causes of mortality include OI-related complications, cardiovascular and respiratory issues, and fracture-related trauma.

The heterogeneous, progressive nature of SSEs supports the need for tailored, multidisciplinary long-term care. However, substantial evidence gaps and methodological inconsistencies limit comparability, highlighting the need for further evidence.

## Highlights

• Evidence is fragmented with varied availability and quality across outcomes.
• Severe osteogenesis imperfecta is often diagnosed earlier than milder disease.
• Symptoms vary by age; some progress rapidly in children, others emerge in adulthood.
• Life expectancy is reduced due to condition-related complications, e.g. heart issues.

## 1 Background

Osteogenesis Imperfecta (OI) is a rare hereditary condition that causes impaired structure of bone and connective tissues, resulting in increased skeletal fragility (Marini et al., 2007). Individuals with OI may also experience a range of associated symptoms, such as hearing loss, growth deficiencies, and cardiovascular abnormalities (Forlino and Marini, 2016). OI is caused by genetic mutations that disrupt the production and structure of collagen (Marini and Do, 2000) and presents with considerable phenotypic heterogeneity classified based on clinical severity (Sillence et al., 1979) and genetic background (Marini et al., 2017). It contrasts more common bone-loss disorders such as osteoporosis, which is characterised by low bone mass and microarchitectural deterioration resulting from cumulative bone remodelling imbalance and aging (van Oostwaard, 2018). Forms of OI that can be identified at birth occur with an incidence of 1 in 10,000–20,000, while mild forms, usually diagnosed later in life, occur with a comparable frequency (Marini and Do, 2000; Bregou Bourgeois et al., 2016a; Folkestad, 2018a; Bishop, 2021).

There are no widely approved treatments for OI; therefore, most treatments are used off-label. Neridronate is the only bisphosphonate nationally approved in Italy for treatment of OI, and pamidronate is the only nationally approved bisphosphonate in Japan for the treatment of OI (Hart et al., 2024; Harada et al., 2020). Treatments for OI can help to improve clinical findings like low bone mass (Hart et al., 2024; Ralston and Gaston, 2019); however, there is no conclusive evidence supporting their effectiveness for fracture prevention in OI (Hald et al., 2015; Dwan et al., 2016). In the absence of effective or curative treatment, a notable unmet need remains (Westerheim et al., 2024), as the recurrence of fractures and other long-term symptoms significantly impact the lives of people with OI (Hart et al., 2024; Hill et al., 2014; van Welzenis et al., 2024). Increased awareness of the wide range of signs and symptoms associated with OI and their progression could help enable improvements in future care (Hart et al., 2024).

The natural history of a condition is best understood through longitudinal, prospective studies that follow its progression (Administration, U.F.a.D, 2019; Liu et al., 2022). This is challenging for OI due to its rarity and phenotypic diversity, which require differing treatments and managements (Pogue et al., 2018; Roughley et al., 2003). While some longitudinal studies have gathered valuable data on OI (Folkestad et al., 2016; Joshi et al., 2023), such data remains sparse. When prospective, longitudinal data are challenging to obtain, retrospective cohort studies and cross-sectional studies, where age is used as an analytical factor, can also provide valuable insights (Critchley, 2004).

Natural history data traditionally describes the progression of a condition in the absence of any intervention (Centers for Disease Control and Prevention (CDC), 1978). However, in conditions such as OI, interventions and treatments are essential and thus administered to almost all patients. As a result, studies providing natural history data on OI typically include people receiving treatment. These studies still provide valuable insights by identifying demographic, genetic, and environmental factors that correlate with the condition (Liu et al., 2022).

Although informative data on OI natural history exists, no systematic review (SR) has been conducted to collate it to date. By including both longitudinal and cross-sectional data where age is used as an analytical factor, this SR aims to offer a synthesis of OI natural history data currently available in its broadest sense, capturing initial presentation and long-term outcomes. It also aims to identify common signs and symptoms, progression patterns, and areas where inconsistencies exist. This could serve as a valuable foundation of knowledge to help enhance care for individuals with OI.

## 2 Methods

This SR was undertaken following the Centre for Research and Dissemination's guidance on conducting systematic reviews (Centre for Reviews and Dissemination, 2009) and the study protocol prospectively registered on PROSPERO (CRD42024536369).

The overarching review question was: What is the natural history of OI?

Sub-questions included the following:

• What is the typical age and presentation at OI diagnosis?
• What is the morbidity associated with OI, including signs, symptoms, and events (SSEs)?
  ○ What is the age of onset of these SSEs?
  ○ How do SSEs relate to OI type and ‘severity’?
• What is the life expectancy of a person with OI?
• What are the causes of mortality in OI?

### 2.1 Search strategy and selection criteria

Following the PICOS principles (O'Connor et al., 2008), inclusion and exclusion criteria used to conduct this SR are listed in Table 1.

**Table 1:** Inclusion and exclusion criteria used for study selection.

Characteristics	Inclusion criteria	Exclusion criteria
Population	People with OI	People without OI Mixed populations (when the population includes individuals without OI)
Interventions	None or any	N/A
Comparators	None or any	N/A
Outcomes	Age of diagnosis SSEs leading to diagnosis referral Natural history data relating to: SSEs Age of onset SSEs specific to sub-populations Life expectancy in years Causes of mortality	Any other outcomes
Study design	Any studies of at least 5 years of data collection, or sub-grouped by age, or using age as an analytical factor, or describing a distinct life stage (e.g. puberty, menopause, pregnancy), including: Cohort studies, including patient registry data Patient surveys Cross-sectional studies Experimental or quasi-experimental studies	All other study designs Studies with fewer than 10 participants
Language	English	Non-English
Publication type and status	Publication type: Full-text manuscripts Conference proceedings limited to the previous three years Publication status: Published In-process E-publications ahead of print	Publication type: Letters Editorials Notes Lectures Reviews, including systematic literature reviews
Data of publication	2004 onwards	Pre-2004

Abbreviations: OI, osteogenesis imperfecta; SSEs, signs, symptoms, and events.

Following a pre-defined literature search strategy (see A.1), the following electronic databases were searched on the 24th of March 2024: MEDLINE (MEDALL), Embase, and Embase Conference Searches. In addition, conference proceedings of five conferences (2021–2024) were searched by hand: OI congress (2022), International Conference on Children's Bone Health (2022), European Calcified Tissue Society (2021−2023), International Osteoporosis Foundation (2021–2024), and The American Society for Bone and Mineral Research (2021–2024).

Following de-duplication, which was performed using EndNote X8 and manual review, titles and abstracts were screened to exclude irrelevant studies. Two reviewers (AR/MR) performed this in parallel, screening all texts manually. Backwards citation searches were performed on all studies from the title and abstract screening stage using the citationchaser tool, an R package and shiny application (van Haastrecht et al., 2021; Haddaway et al., 2022). Full-text articles of the remaining studies were then reviewed for eligibility. Disagreements between the two reviewers were resolved through discussion or, where necessary, by consulting a third reviewer to reach a consensus.

### 2.2 Data extraction

Two reviewers (AR/AS) extracted information from all relevant included studies, including article details, regions, study design, treatment centre, sample size, patient demographics, and natural history data. All data extraction was manually checked for errors by a single researcher (AR).

### 2.3 Data synthesis

Due to heterogeneity between study methods and populations, data were synthesised narratively, and quantitative values were included only when they were clearly defined and explicitly stated in the original study. Data were grouped by SSEs associated with OI, and by data types (retrospective or prospective, and longitudinal, cross-sectional, or survey-based). The synthesis aimed to identify patterns in OI progression and differences in outcomes between key demographics such as age groups, sex, and OI type, where this was specified.

### 2.4 Quality assessment

One researcher (AR) performed quality assessment alongside data extraction. We used various bespoke versions of the JBI Case Series Critical Appraisal Tool (Critical Appraisal Programme, 2023), the CASP Cohort Study Checklist, and the JBI Cross Sectional Critical Appraisal Tool (see A.2) (Moola et al., 2017).

## 3 Results

**Fig. 1:**
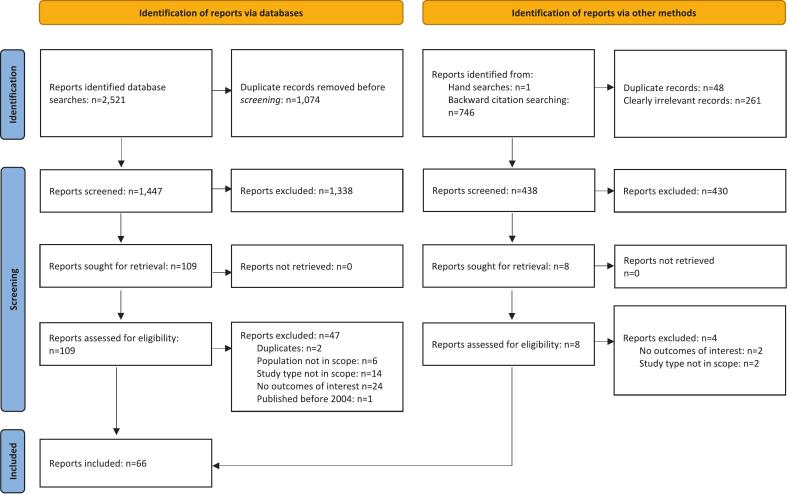
PRISMA flow chart of study selection process Abbreviations: PRISMA, Preferred Reporting Items for Systematic reviews and Meta-Analyses.

This review included 66 unique studies (Fig. 1) covering diagnosis of OI (*n* = 13), progression of SSEs (*n* = 51), and mortality (*n* = 3). Forty-four reports included longitudinal analyses, and 29 included cross-sectional analyses with age as an analytical factor; some included more than one type of analysis. The median cohort size was 84 individuals, ranging from 12 to 8444. Most studies did not distinguish between OI types; three focused on Type I and three on OI severity (Table 2; see A.3 for detailed study's characteristics).

### 3.1 Study quality assessment

The quality of the 66 studies included in this review was variable, with some lacking demographic and clinical information. For example, 49 studies did not report the ethnic backgrounds of the cohorts. Four case series and four cross-sectional studies did not provide details such as OI type distribution, mean age, or sex distribution, which are important for drawing conclusions about their findings or making comparisons with other studies. Handling of confounding factors was another concern, with five cohort studies and nine cross-sectional studies providing insufficient strategies to address them. Furthermore, four studies were limited to abstracts, making it challenging to fully assess their quality and reliability (see A.2 for details).

### 3.2 Typical age and presentation at OI diagnosis

**Fig. 2:**
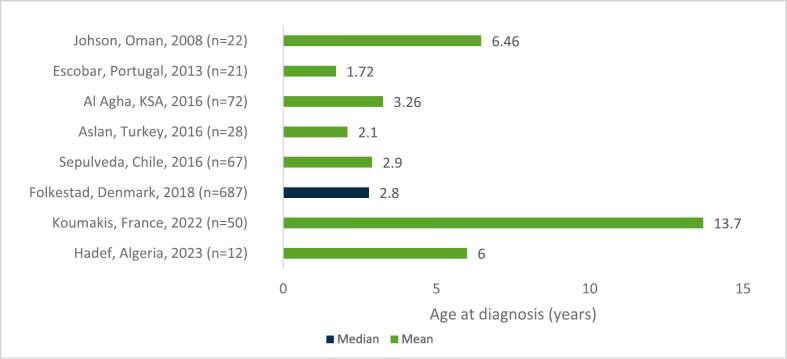
Mean or median age at diagnosis reported in *n* = 8 studies Notes: Only studies where the entire observed cohort are included in the average are included in this figure. Abbreviations: KSA, Kingdom of Saudi Arabia.

Twelve studies reported age at OI diagnosis from longitudinal analyses (Aslan et al., 2016; Corio, 2023; Escobar et al., 2013; Folkestad, 2018b; Hadef et al., 2023; Johnson and Varghese, 2008; Caudevilla Lafuente et al., 2020; Greeley et al., 2013; Al-Agha et al., 2016; Koumakis et al., 2022; Schramm et al., 2009; Sepulveda et al., 2017). Eight of these studies reported an average age of diagnosis across their entire observed cohort, which ranged from 1.7 to 13.7 years (Fig. 2). The highest average age at diagnosis across the entire observed cohort was reported by Koumakis et al. (2022), who found an average of 13.7 years in a cohort of previously pregnant women, a group likely to have milder phenotypes than the general OI population (Koumakis et al., 2022). Within studied subgroups, severe OI phenotypes were often diagnosed at birth or in utero, while mild OI diagnoses were often made in infancy. In some instances, OI can remain undiagnosed until adulthood, with Caudevilla Lafuente et al. (2020) reporting an average age of diagnosis of 35.6 among an adult subpopulation (*n* = 11) (Caudevilla Lafuente et al., 2020).

**Table 2:** Characteristics of included records (*n* = 66).

Category	n (%)
Publication year	
2004–2008	7 (11)
2009–2012	9 (14)
2013–2016	14 (21)
2017–2020	18 (27)
2021–2024	18 (27)

Publication type	
Journal article	61 (92)
Pre-print	1 (2)
Abstract only	2 (3)
Journal article with only abstract in English	2 (3)

Temporal design^a^	
Prospective	21
Retrospective	48

Study design^a^	
Longitudinal	44
Cross-sectional with age as an analytical factor	29

Study population^b^	
Children and adults with OI	35 (53)
Adults with OI	4 (6)
Women with OI	4 (6)
Children with OI	21 (32)
Neonates/foetuses with OI	2 (3)

Data source	
Medical records only	41 (62)
Medical examination only	15 (23)
Medical examination and records	8 (12)
Survey data	2 (3)

Source of study population^c^	
Single centre (generalised)	23 (35)
Single centre (specialised)	20 (30)
Multiple centres	7 (11)
National registries	15 (23)
Court proceedings	1 (2)
Not reported	1 (2)

Geographical region	
North America	20 (30)
Europe	27 (41)
Asia	11 (16)
South America	5 (8)
Africa	1 (2)
Oceania	1 (2)
International	1 (2)

Abbreviations: OI, osteogenesis imperfecta.

a Some studies included data generated with more than one temporal and/or study design.

b The definitions of “children” and “adults” varied across studies included in this table, and these classifications were not systematically defined. In this review, we define children as those under 18 years old, though some studies included in this review may include young adults in their paediatric cohort.

c Generalised single centres include non-specialist hospitals and departments based in general hospitals (e.g. endocrinology departments). Specialised centres include any facilities explicitly reported to specialise in the treatment of OI, orthopaedic hospitals or tertiary centres. National registries include OI patient databases in a single country.

Greeley et al. (2013) reported quantitative data on typical SSEs present at diagnosis. They reported the number of fractures found at the time of diagnosis in children and infants, showing that all individuals with three or more fractures at presentation were diagnosed prenatally. None of the individuals diagnosed within the first year of life in the study had more than two fractures at the time of diagnosis (Greeley et al., 2013).

### 3.3 Morbidity associated with OI, including signs, symptoms, and events

#### 3.3.1 Musculoskeletal problems and physical impairments

Twelve studies reported on age at first fracture. Average age at first fracture ranged from 0.96 to 2.63 years old where data was reported across the entire observed cohort (Wei et al., 2022; Xi et al., 2021), whereas averages from subgroup analyses varied from 0.03 to 11.5. Children with severe OI types generally fractured the earliest, with Wei et al. (2022) finding average ages of 1.50, 0.03 and 1.25 years old for children with OI Type I, III, and IV respectively (Wei et al., 2022). Across OI types, children often fractured in the first six months of life (De Wouters et al., 2022; Brizola et al., 2014), with fractures at birth common, especially in children with Type III OI (De Wouters et al., 2022; Brizola et al., 2014). First fractures commonly occurred in limbs, though vertebral fractures, were also common at diagnosis (Greeley et al., 2013; Sepulveda et al., 2017; Binh et al., 2017).

Six studies reported on fracture rates and location throughout life (Joshi et al., 2023; Greeley et al., 2013; Koumakis et al., 2022; Sepulveda et al., 2017; Folkestad et al., 2017; Bains et al., 2019). On average, children fractured more frequently than adults (Joshi et al., 2023; Folkestad et al., 2017; Bains et al., 2019). For children with Type I OI, Joshi et al. (2023) found the highest rate (469 fractures/1000 patient-years) occurring under age 3 years, with rates declining with age (down to 140 fractures/1000 patient-years at ages 15–18). Limbs were the most common fracture location among children. Furthermore, they found, 14.7% of fractures were in the same anatomical site as a previous fracture (Joshi et al., 2023). Among adults, individuals under 20 years had the highest fracture rate, followed by an increase in fracture rates after age 55, particularly in women (see A.4) (Folkestad et al., 2017; Bains et al., 2019). Fractures of the hips and femur were more common in those over 55 years old, especially in women (Folkestad et al., 2017).

Koumakis et al. (2022) focussed on pregnancy-related fractures, finding 24% of women experienced fractures during pregnancy, most frequently in the third trimester, or in the 6 months following delivery, with an average delay of 2 months post-delivery. Most fractures (69%) were not caused by direct trauma. Similar fracture locations, including the hips and femoral neck, were noted in this group (Koumakis et al., 2022). All women who had post-partum fractures (8/8, 100%) were breastfeeding, compared with about half of women breastfeeding without fractures (25/53, 47.1%; (*p* = 0.006). (Koumakis et al., 2022).

Six studies reported on scoliosis (Bains et al., 2019; Anissipour et al., 2014; Wei et al., 2012; Watanabe et al., 2007; Chen et al., 2023; Darba and Marsa, 2020). One study found an average diagnostic age of 7 years, but many children had developed curvature earlier than recorded (Anissipour et al., 2014). Scoliosis prevalence increased with age, affecting 0.85% of children under 2 years old versus 15.31% in adults aged 18–40 (Darba and Marsa, 2020). In one study, 50% of children had scoliosis (Anissipour et al., 2014). Scoliosis worsened faster in children with Type III and IV OI than in Type I (Bains et al., 2019; Anissipour et al., 2014).

Four studies reported on cranial junction problems (CJP). Basilar impression was on average diagnosed at 2 years old, with 80% of diagnoses made by age 20 (Arponen et al., 2012) and could either resolve or worsen over time (Cheung et al., 2007). Cheung et al. (2011) found that 4% of children had basilar invagination, but hypothesised that this number would be larger in older populations, as basilar invagination predominantly develops during adulthood (Cheung et al., 2007). Platybasia and Wormian bones were typically diagnosed at birth and remained permanent conditions (Cheung et al., 2007; Semler et al., 2010). Arponen et al. (2015) investigated risk factors for CJP and identified severe OI as a risk factor for basilar impression or invagination (OR = 22.04) (Arponen et al., 2015).

Six studies reported on other bone-related problems (Brizola et al., 2014; Ozturk et al., 2022; Kok et al., 2013; Edouard et al., 2011a; Edouard et al., 2011b; Bobak et al., 2024). Lumbar spine BMD *Z*-scores increased with age, showing earlier improvements in girls compared with boys. Children with OI Type III consistently had the lowest Z-scores, indicating significantly reduced BMD compared to the average for their age group (see A.5) (Ozturk et al., 2022; Kok et al., 2013). The authors noted that most children included in these studies were receiving bisphosphonate treatment, which likely affected BMD (Ozturk et al., 2022; Kok et al., 2013). One study found that 27% of participants were deficient in vitamin D; increasing age, season, and OI severity were positively correlated with vitamin D deficiency (*p* < 0.001 for age and season; *p* = 0.048 for OI severity) (Edouard et al., 2011a). In one study, the relative risk of osteomyelitis following surgery was over 30 in children, compared to 6 in adults (Bobak et al., 2024). Another found that bone deformities were associated with having experienced over 10 fractures, a delayed gait onset and muscle weakness in children with OI (Brizola et al., 2014).

Three studies examined joint issues linked to OI (Ahn et al., 2019; Andersen et al., 2022; Mei et al., 2024). One found an increase in acetabular protrusion from 45% at the first assessment to 66% at 2-year follow-up in a cohort of all ages (Ahn et al., 2019). Predictive risk factors included female sex, older age, higher BMI, and contralateral acetabular protrusion. Another study found that osteoarthritis often develops before age 40 in people with OI (Andersen et al., 2022). Generalised hypermobility was found in 33% of children (*n* = 25) and 20% of adults (*n* = 23) with OI; peripheral joints were most affected (Mei et al., 2024).

Seven studies investigated growth issues in children with OI (Darba and Marsa, 2020; Ozturk et al., 2022; Nicol et al., 2021; Yimgang and Shapiro, 2016; Graff and Syczewska, 2017; Barber et al., 2019; Germain-Lee et al., 2016). Two studies provided growth curves showing that growth in children with OI was slower than in the general population, particularly during the pubertal growth spurt and in those with severe OI types (Graff and Syczewska, 2017; Barber et al., 2019). In one of these studies, BMI remained comparable with the general population at all ages (Graff and Syczewska, 2017). Another study found that height *Z*-scores were lower in adults compared with children, with the decline being more pronounced in severe OI types (see A.6) (Germain-Lee et al., 2016).

Ozturk et al. (2022) considered pubertal features in children with OI, noting an increase in obesity at the onset of puberty (from 6 to 17.2%) (Ozturk et al., 2022). Precocious puberty predominantly affected boys, and was only observed in children who were obese (Ozturk et al., 2022). Age at menarche was consistent with the general population (Ozturk et al., 2022; Yimgang and Shapiro, 2016). One study found that the association between collagen X biomarker (CXM) levels and growth velocity was weaker in children with OI than in the control population (Nicol et al., 2021).

Three studies investigated mobility problems (Brizola et al., 2014; Rodriguez Celin et al., 2023; Machol et al., 2020). Age at walking onset was delayed in those with OI compared to the general population, and children with OI Type III were most affected (Brizola et al., 2014; Machol et al., 2020)(see A.7). Age at first walk was delayed compared to general populations by 3-months and 33-months on average for Type I and Type III respectively, for those who eventually walked (Machol et al., 2020). Height was identified as a significant predictor of mobility outcomes, while weight, age, and bisphosphonate history were not significant predictors (Machol et al., 2020).

One study examined chronic pain, defined as pain lasting more than 3 months, which affected 41.8% of the cohort compared with 20% in the general population (Rodriguez Celin et al., 2023; van Hecke et al., 2013). Within this cohort, adults were more affected than children (69.7% vs. 29.7%). Predictors of chronic pain were age, wheelchair use, and frequent fractures. Being female and having higher body weight were additional predictive factors for those with OI Type I.

#### 3.3.2 Cardiovascular problems

**Fig. 3:**
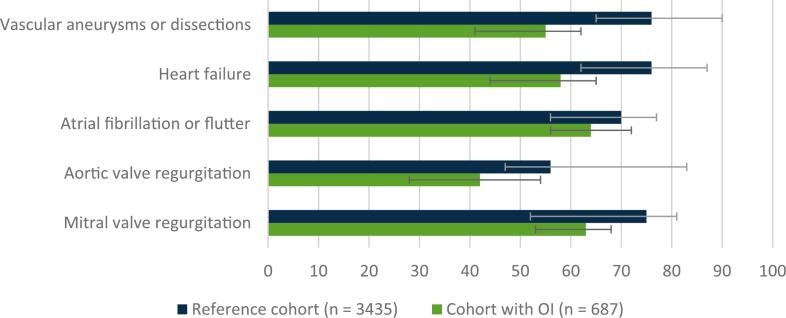
Median age of onset of cardiac issues in people with OI compared with a reference population Notes: Adapted from Folkestad et al. (2016).

Four studies examined cardiac issues in people with OI (Folkestad et al., 2016; Darba and Marsa, 2020; Mei et al., 2024; Radunovic and Steine, 2015). Mild valve regurgitation was the most common issue at all ages (Mei et al., 2024; Radunovic and Steine, 2015). Folkestad et al. (2016) reported statistically significant rises in the incidence of heart-related conditions compared to the general population—specifically heart failure, mitral valve regurgitation, aortic valve regurgitation, and atrial fibrillation or flutter—the onset of which typically occurred earlier than in the general population, at around the age of 50 (Fig. 3) (Folkestad et al., 2016).

Children with OI showed better systolic function than adults, as seen in the general population (Mei et al., 2024); however, parameters differed for individuals with OI compared with the general population when adjusted for body surface area (BSA), as indicated by lower fractional shortening per BSA in children (33.95% vs. 25.63%, *p* = 0.024) and higher left ventricular ejection fraction per BSA in adults (43.55% vs. 37.98%, *p* < 0.001) (Mei et al., 2024). Left ventricular function decline was associated with increasing age, male sex, and increased blood pressure, while right ventricular function was associated with OI type, with more severe phenotypes being significantly associated with several indicators of worse right ventricular function (*p* < 0.05) (Radunovic and Steine, 2015). One study found a rise in hypercholesterolaemia and hyperlipidaemia diagnoses after age 40, which can be associated with cardiac complications (Darba and Marsa, 2020).

Two studies considered breathing issues in OI (Caudevilla Lafuente et al., 2020; Tam et al., 2018). Individuals with Type III OI have significantly reduced FEV1 and FVC compared to general populations, indicating worse lung performance (Tam et al., 2018). Chest wall deformities primarily affected those with severe OI (according to the Van Dijk and Sillence clinical classification (Van Dijk and Sillence, 2014)) and further reduced lung function in these individuals (Caudevilla Lafuente et al., 2020).

#### 3.3.3 Reproductive problems

Four studies investigated pregnancy outcomes in women with OI (Koumakis et al., 2022; Yimgang and Shapiro, 2016; Yimgang et al., 2016; Rao et al., 2021). One reported that 12 out of 50 women experienced pregnancy- or lactation-related fractures (25% vertebral and 30% femoral). Those who fractured were, on average, older during pregnancy (32.7 vs. 29.3, *p* = 0.002), and breastfed more frequently (85.7% vs. 47.1%, *p* = 0.03) (Koumakis et al., 2022). Another study found a significantly higher fracture risk in the OI population compared to the general population during and after pregnancy (RR 221, *p* < 0.001) (Rao et al., 2021). One study found no significant difference in age at menopause between OI cohorts and the general population (Yimgang et al., 2016).

Regarding neonatal outcomes, one study reported that neonates born to women with OI had a higher risk of low birth weight (<2500 g; 26.2% vs 6.8%, p < 0.001), and higher rates of neonatal intensive care unit (NICU) admissions (19% vs. 5.68%, p < 0.001) compared to the general population (Rao et al., 2021). NICU admissions primarily followed respiratory issues and fractures (Yimgang et al., 2016).

#### 3.3.4 Dental, sight, and hearing problems

**Fig. 4:**
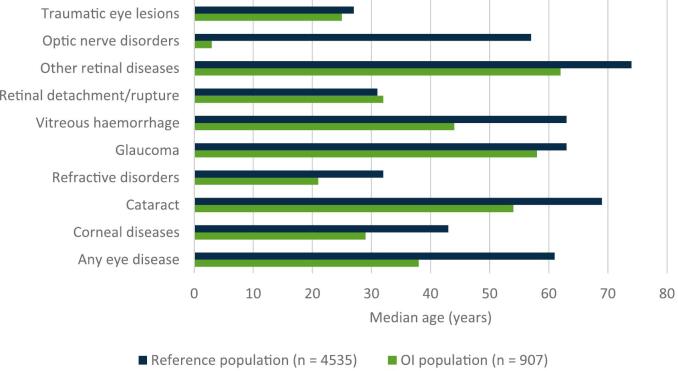
Median age of onset of eye problems in OI cohort compared with a reference population Notes: Adapted from Lyster et al. (2022).

Lyster et al. (2022) reported that individuals with OI (*n* = 907) had a significantly increased risk of developing eye diseases (IR = 4.07, 95% CI = 3.41–4.85), and that they typically developed them at an earlier age than the general population (Fig. 4) (Lyster et al., 2022).

Eight studies examined hearing loss in individuals with OI (da Costa Otavio et al., 2020; Ltaief-Boudrigua et al., 2022; Martens et al., 2018; Pillion and Shapiro, 2008; Swinnen et al., 2011; Waissbluth et al., 2020; Swinnen et al., 2012). Waissbluth et al. (2020) reported that people with hearing loss were, on average, older than those without (36.2 vs. 24.2 years) (Waissbluth et al., 2020), while Swinnen et al. (2011) noted that 34.1% of adolescents with OI already had hearing loss (Swinnen et al., 2011). Conductive hearing loss was more common in children, while sensorineural or mixed types became more prevalent with age (da Costa Otavio et al., 2020; Pillion and Shapiro, 2008).

Two studies found a similar prevalence of dentinogenesis imperfecta in adults and children with OI, and that those with Type III OI were most affected (Arponen et al., 2015; Mei et al., 2024). Children who had never been treated with bisphosphonates were also found to have an earlier age of tooth eruption than the general population (Arponen et al., 2015).

### 3.4 Life expectancies and causes of death of people with OI

**Fig. 5:**
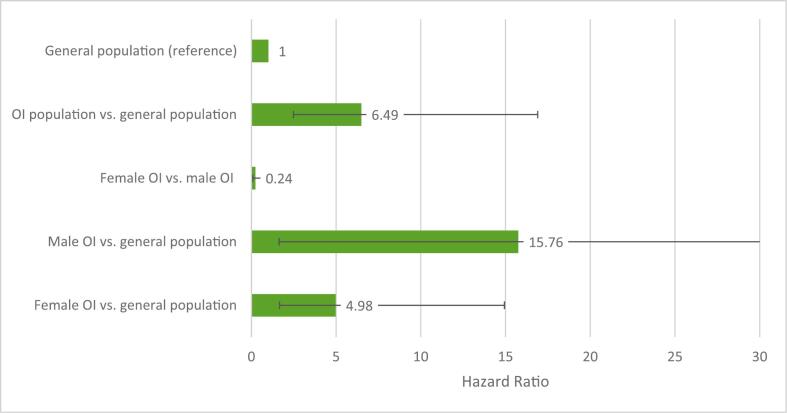
Gender-specific mortality risks in a cohort with OI (*n* = 319) compared to the general population, with 95% confidence intervals indicated by error bars (Corio, 2023). The upper 95% CI for male OI vs general population is 151.57.

Three studies examined mortality in individuals with OI, finding a slightly reduced life expectancy compared with the general population (Corio, 2023; Folkestad, 2018b; Gimeno-Martos et al., 2017). One study reported a median survival age of 72.4 vs. 81.9 for men and 77.4 vs. 84.5 for women. This study also reported 26 child deaths (15 before 1 year of age) and 4.5% of deaths due to external causes, including trauma and fractures (vs. 1.9% in the general population) (Folkestad, 2018b). Although cardiovascular disease was a frequent cause of death in individuals with OI, there was no increased relative risk (RR) compared to the reference population in this study (Folkestad, 2018b). Additionally, there was an increased RR of death in the OI population due to increased sub-hazard ratios (SHR) of respiratory disease (SHR 3.1 [95% CI: 1.4–6.9]) and gastrointestinal disease (SHR 4.2 [95% CI: 1.6–10.8]) (Folkestad, 2018b). Another study found a 2.8% mortality rate with a hazard ratio (HR) of 6.49 for OI, with men at higher risk (HR 15.76) than women (HR 4.98) (Fig. 5) (Corio, 2023). The leading causes of death included OI (20%), which was not further defined, and cardiovascular disease (20%). A third study reported a 6.2% mortality rate over 17 years in a cohort of hospitalised individuals with OI, with an average age of death of 60.8 years (Gimeno-Martos et al., 2017).

## 4 Discussion

This SR synthesises literature on the natural history of OI in contemporary cohorts, many of which receive treated patients, revealing variable diagnostic patterns and an understanding of the condition's progression that is limited by a lack of prospective longitudinal data. Key findings include an earlier onset and increased frequency of many SSEs compared with the general population. Additional findings were age- and OI severity-related increases in the impact of several signs and symptoms and a slightly reduced life expectancy. The findings in this report were limited by challenges caused by heterogeneous study designs, outdated or limited datasets, and a lack of comprehensive data on specific outcomes like mortality causes. Study quality was also variable and limited the interpretation of findings. Despite these limitations, our broad inclusion of cross-sectional data where age was an analytical factor enabled us to highlight important patterns and gaps, supporting the need for further longitudinal research. To best represent the OI population, this review included any type of intervention, as it is challenging to identify data on completely untreated individuals due to the importance of treatment in a highly burdensome disease. Identified studies included the use of treatments in most patients, with a wide variation in treatments and interventions among individuals depending on their signs and symptoms and medical history.

In this review, the age at diagnosis varied between studies, however, was reported to occur on average in childhood, and at younger ages in Type III OI compared to Type I OI. Studies showed that there can be a significant delay between the onset of signs and symptoms—notably fractures—and OI diagnosis. This was frequently observed in people with Type I OI, where symptoms are often milder than for those with more severe types (Brizola, 2017). Some individuals do not receive a diagnosis until their own children present symptoms and receive a diagnosis (Osteogenesis Imperfecta Foundation, 2024). Studies often focused on outcomes other than diagnosis and reported diagnosis as contextual information rather than a primary outcome, limiting our ability to establish a clear and comprehensive understanding of diagnosis of OI.

Overall, cohort sizes and characteristics varied across studies, making it difficult to draw consistent conclusions on the age at diagnosis of OI. Additionally, one of the primary challenges in understanding when people with OI receive diagnosis is that many available data are outdated, with some collected decades ago. These data may not represent current trends, as the clinical knowledge surrounding OI has since improved and diagnostic tools are more readily employed (Bonafe et al., 2015; van Dijk et al., 2012; Trejo and Rauch, 2016; Groft et al., 2021). Diagnosis typically involves a combination of medical history, clinical examination, genetic testing and radiographic imaging. (Trejo and Rauch, 2016; Bregou Bourgeois et al., 2016b). Some OI diagnoses can be reliably made as early as the first to early second trimester of pregnancy; however, this is most typically the case for severe forms of OI, which often cause visible bone deformities in utero (Thompson, 1993), and may be more likely in the case of family history of OI.

One key reason for delayed diagnosis is that early fracture-related symptoms in milder OI types can resemble the effects of non-accidental injury or other medical conditions, such as Ehlers-Danlos syndrome (Kocher and Dichtel, 2011; Arundel and Bishop, 2010; Miklovic and Sieg, 2023). Moreover, healthcare professionals may lack sufficient awareness of the condition, which could prevent them from identifying specific clinical patterns that might prompt investigation (Kocher and Dichtel, 2011; Arundel and Bishop, 2010). Consequently, individuals may not receive timely symptom management. The long-term consequences of delayed treatment remain poorly understood due to limited research.

In this review, we were unable to comprehensively capture the typical presentation of OI at diagnosis. This limitation arises from our inclusion criteria, which were designed to collate natural history data with a focus on longitudinal outcomes and age-related progression. As a result, qualitative descriptions of specific SSEs at the time of diagnosis, such as certain types of bone deformities and blue sclerae, were not included (Weaver et al., 2021). While these SSEs are recognised as indicators of OI, their absence in longitudinal datasets may reflect a gap in the current literature that warrants further investigation.

OI causes a wide range of signs and symptoms that affect overall development and functioning, resulting in a significant burden on affected individuals and caregivers. Studies included in this review provide an overview of the range of SSEs experienced by people with OI, as well as evidence of considerable heterogeneity in the presentation and evolution of the condition between individuals. People with severe OI tend to experience SSEs earlier and more intensely than those with mild OI. However, they can still experience similar SSEs throughout the course of their lives.

Most SSEs appeared earlier than in the general population, but data comparing symptoms prevalence and severity across lifetime are limited other than fractures. The severity of some symptoms was shown to increase with age, as they did not typically resolve. A better understanding of the progression of SSEs can enable early identification and better preventive care.

While this review did not systematically evaluate the link between signs and symptoms, and causation is difficult to assess through observational studies, we expect that SSEs will be interlinked in terms of exacerbations and combined impacts on patient holistic health. The wider literature is supportive of this hypothesis. For example, obesity through adulthood has been linked with an increase in the number of osteoporotic fractures with a slower recovery period in otherwise healthy individuals (Shapses et al., 2017). Pain is also a significant issue for people with OI, which may compound other risks. For example, one study found that baseline pain at any location increased the likelihood of experiencing falls in the following year in older men, indicating how certain SSEs can be interlinked (Munch et al., 2015). Another study found that initial fractures before the age of two, and a high number of lower extremity fractures were both associated with decreased physical function in adulthood. (Matsushita et al., 2020) However, quantitative data supporting relationships across SSEs is currently lacking, and further research would be needed to characterise these relationships in OI.

The lack of longitudinal data on SSEs stems primarily from the rarity of OI, which complicates efforts to conduct long-term studies. Tracking the progression of SSEs over decades requires substantial resources and ongoing participant retention, both of which are challenging (Adang, 2024). Additionally, there is a separation between paediatric and adult care in the management of OI, which often fragments medical records (Rauch and Morin, 2023). Compounding this structural issue, many patients are lost to follow-up during the transition to adult care; in one study, fewer than half remained in established follow-up three years post-transfer (Wright et al., 2024). This lack of longitudinal insight complicates long-term care planning for OI, as the diverse and evolving nature of the condition suggests that a personalised, multidisciplinary care approach could be especially beneficial (Monti et al., 2010).

Life expectancy is lower in people with OI than in the general population, however, current evidence is lacking. Both men and women appear to live to ages comparable with the general population (Corio, 2023). Data on causes of death are limited and often lack specificity. Available evidence indicates that common causes include external trauma and cardiovascular complications; one study suggested that OI itself is a major cause of death (Corio, 2023). This lack of specificity highlights the need for further investigation into the mortality patterns associated with OI.

The data in this review on mortality did not distinguish between OI types, though prior research shows that life expectancy varies significantly. Individuals with OI Types I and IV generally have a life expectancy close to that of the general population. They often die of causes unrelated to OI, whereas those with Type III face a reduced life expectancy and higher risks of OI-related complications (Corio, 2023; McAllion and Paterson, 1996). Additionally, the studies reviewed are from countries with advanced healthcare systems, such as Denmark and Taiwan. This limits the generalisability of findings, as access to treatment for rare conditions varies across regions (Rajtar and Knoll, 2024).

The registry-based studies reporting mortality in this review had small cohorts, which can be explained by the rarity of OI, leading to broadly grouped or omitted data for anonymity. Large-scale, longitudinal studies tracking SSE progression in older adults could better clarify mortality risks and differences across OI severities.

### 4.1 Strengths and limitations of this SR

This SR provides an overview of the current knowledge surrounding the natural history of OI. The systematic approach minimised bias and revealed significant knowledge gaps. By encompassing all outcomes and OI types, we were able to present a nuanced view of the condition's natural history, including both long-term progression and short-term events such as puberty and pregnancy. However, whilst we captured natural history data using as broad a scope as possible, this review was not suited to describe the typical presentation of OI at the time of diagnosis, due to the qualitative nature of the data regarding this outcome in the literature.

This review highlights the overall lack of longitudinal prospective data on OI, which best characterises natural history outcomes. However, our inclusion of cross-sectional data with age-related outcomes enabled a broader characterisation of the condition's presentation and progression. Despite our broad range of included studies, some outcomes, such as digestive or tendon issues, were not found in our search. This could stem from the absence of age-related data on these outcomes. Our findings were weighted toward North America and Europe, representing a geographical bias. This may in part be due to the English-language inclusion criteria, but may also represent geographical gaps in the literature.

There were some limitations to the data quality and reliability across the literature in the context of natural history. Due to anticipated evidence gaps, we chose to include all studies regardless of quality assessment outcomes; while this approach minimised the risk of evidence gaps, it may compromise the strength of findings. The heterogeneity of the included studies, which varied in design, population, and outcome measurements, as well as differences in how factors such as OI severity and type are reported, hindered the comparability of findings. Additionally, due to the importance of treatment for long-term outcomes for people with OI, many included studies observed individuals receiving interventions, impacting the ability to understand the progression of outcomes in entirely untreated individuals. Treatment status, intensity, and duration were not consistently reported across studies, limiting opportunities to conduct stratified synthesis. This may have introduced some variation in results between studies and individuals.

## 5 Conclusion

This SR highlights both the complexity of OI and paucity of literature on OI natural history; whilst highlighting the high burden that OI causes to patients and caregivers. While aspects of the range of effects of this condition are well characterised in current literature, significant gaps in knowledge surrounding the lifelong progression of SSEs remain. Variability in symptom presentation across OI types complicates the characterisation of the natural history of OI, as individuals with the same condition can experience widely varying symptom severities; this challenge is underlined by the difficulties in diagnosing OI. The studies included in this review suggest that a multidisciplinary approach aimed at reducing complications and improving long-term outcomes could support the management of people with OI. To better understand the progression and variability of OI, further longitudinal, prospective studies are essential. The Brittle Bone Disorders Consortium (BBDC), part of the Rare Diseases Clinical Research Network, is an important initiative contributing to longitudinal natural history studies and improved standards of care (Brittle Bone Disorders Consortium, 2023). Other ongoing efforts includes the SATURN programme, which aims to integrate existing and prospective data into a unified core dataset (Sangiorgi et al., 2024; Juarez et al., 2025) and the Key4OI registry and the OI module within the European Registry for Rare Endocrine and Bone Conditions which also seek to improve the accessibility of registry data in OI (Ali et al., 2021; Key4OI, 2025). Furthermore, collaborations such as the Pain Project are focused on expanding knowledge of symptoms that remain poorly characterised in the literature (Osteogenesis Imperfecta Foundation, 2024). Collectively, these initiatives are important steps toward closing evidence gaps within the current literature.

## CRediT authorship contribution statement

**Davide Gatti:** Writing – review & editing, Validation, Supervision. **Samantha Prince:** Writing – review & editing, Validation, Supervision, Conceptualization. **Ogün Sazova:** Writing – review & editing, Validation, Supervision. **Clive Whitcher:** Writing – review & editing, Validation, Supervision, Project administration. **Antoinette Randet:** Writing – original draft, Visualization, Methodology, Investigation, Formal analysis, Data curation. **Matthew Carter:** Writing – review & editing, Validation, Project administration. **Maria Rapoport:** Writing – review & editing, Validation, Supervision, Project administration, Methodology. **Oliver Semler:** Writing – review & editing, Validation, Supervision.

## Ethics approval and consent to participate

Not applicable.

## Funding

Funding for this research was provided by Mereo BioPharma Group, London, United Kingdom.

## Declaration of competing interest

The authors declare the following financial interests/personal relationships which may be considered as potential competing interests: Matthew Carter reports financial support and article publishing charges were provided by Mereo BioPharma Group PLC. Davide Gatti reports a relationship with Mereo BioPharma Group PLC that includes: consulting or advisory. Oliver Semler reports a relationship with Amgen Inc. that includes: consulting or advisory. Oliver Semler reports a relationship with BioMarin Pharmaceutical Inc. that includes: consulting or advisory and speaking and lecture fees. Oliver Semler reports a relationship with Mereo BioPharma Group PLC that includes: consulting or advisory and speaking and lecture fees. Oliver Semler reports a relationship with Kyowa Kirin Co Ltd. that includes: speaking and lecture fees. Oliver Semler reports a relationship with Pfizer Inc. that includes: travel reimbursement. Oliver Semler reports a relationship with Ultragenyx Pharmaceutical Inc. that includes: funding grants. If there are other authors, they declare that they have no known competing financial interests or personal relationships that could have appeared to influence the work reported in this paper.

## Data Availability

Relevant data is provided within the manuscript or supplementary information files.

## Appendix A Supplementary data

Appendix A.1 Search strategies

Appendix A.2 Study quality assessment

Appendix A.3 Detailed summary of included studies with their characteristics.

Appendix A.4 Total number of fractures in OI cohort over 17.9-year observation period, by sex

Appendix A.5 Median BMD Z-score in cohort of 83 children with OI

Appendix A.6 Median height Z-score in children and adults by OI type

Appendix A.7 Age and gait onset by OI type
