# Supplementary material for: The natural history of osteogenesis imperfecta: a systematic review
Source: Bone Rep. 2026 Jun 5;29:101927. doi: 10.1016/j.bonr.2026.101927 (PMC13266223; doi:10.1016/j.bonr.2026.101927)
Supplement: Appendix A.1 — Search strategies [file mmc1.docx]

Appendix A.1. Search strategies

Search strings used to search MEDLINE (via Ovid), on 24 March 2024 (date limit 1946–current)

| 1 Osteogenesis imperfecta/ or (Osteogenesis imperfecta or (brittle adj3 bones)).ti,ab,kf. (6750) | Condition terms: The search opens at Line 1 with the controlled indexing (MeSH) term for osteogenesis imperfecta (the / indicates that this is an indexing term).  We also utilise free-text terms. These are terms identified by the research team. They seek to cover deficiencies in indexing, to identify new or unindexed records, and to search in content fields beyond indexing.  This search uses terms for both osteogenesis imperfecta or brittle bones, as variation in naming is noted in registry reports (c.f. [114] or [115])  We do not distinguish between or define types of osteogenesis imperfecta (e.g. Type 1 etc.) as the underlying condition term does not change by type ( c.f.[116-118] that is, there is not a different sub-name by type, as is the case for some conditions).  Free-text terms are searched in the following fields:   - ti = title - ab = abstract - kf = author generated keyword filed (these are terms chosen by authors to describe their papers)   We use proximity markers (e.g. adj3), allow us to search between search terms to a gap of one space and with the terms in either order. So we will identify ‘brittle leg bone’ or ‘bones were brittle’.  We checked the search strategies and studies included or excluded in the two Cochrane reviews focused on osteogenesis imperfecta. [119, 120] The aim was to critique our draft condition search terms, to see if any terms had been overlooked. We also scoped the literature and studies. This process did not lead us to alter our terms. |
| --- | --- |
| 2 Natural histor*.ti,ab,kf. (57155) 3 Osteogenesis Imperfecta/hi or History.ti. (101029) 4 ((patient* or client or after or provid* or family or families) adj3 histor*).ti,ab,kf. (169888) 5 2 or 3 or 4 [1. terms for natural history or historical data] (298105) | In this section of the search, we are aiming to identify natural history studies, or studies that report longitudinal data.  There are no existing search filters, nor is their guidance on searching for natural history studies or data specifically. So in this aspect of the search, we search in three ways.  1. terms for natural history or historical data  Lines 2-4 describe terms for natural history. We search for this phrase explicitly but also used truncation to pick up plurals. The MeSH term is re-used from Line 1 (above) but focused down using a floating subheading to identify historical data specifically (Line 3). NB: we do not use the MeSH heading for natural history since this is meant for the classification of natural objects. |
| 6 *Retrospective Studies/ (561) 7 (retrospective* adj2 (stud* or review* or analysis or analysed)).ti,ab,kf. (733108) 8 chart review*.ti,ab,kf. (55973) 9 (Medical record* or patient chart*1 or patient record* or registry or registries or claims data* or patient survey*).ti,ab,kf. or *Registries/ or *"Surveys and Questionnaires"/ (435568) 10 *Prospective Studies/ (486) 11 (prospective* adj2 (stud* or review* or analysis or analysed)).ti,ab,kf. (434772) 12 *Case-Control Studies/ (1390) 13 case control*.ti,ab,kf. (166478) 14 *Longitudinal Studies/ (1963) 15 (longitudinal or longitudinally).ti,ab,kf. (368118) 16 *Cross-Sectional Studies/ (571) 17 (cross section or cross sectionally).ti,ab,kf. (35316) 18 *Interrupted Time Series Analysis/ (180) 19 time series.ti,ab,kf. (49515) 20 *Cohort Studies/ or *Birth Cohort/ (1615) 21 cohort*.ti,ab,kf. (928701) 22 Osteogenesis Imperfecta/ep [Epidemiology] (132) 23 (epidemiology or epidemiological).ti,ab,kf. (457897) 24 *Incidence/ or Incidence.ti,ab,kf. (950673) 25 *Prevalence/ or Prevalence.ti,ab,kf. (830899) 26 6 or 7 or 8 or 9 or 10 or 11 or 12 or 13 or 14 or 15 or 16 or 17 or 18 or 19 or 20 or 21 or 22 or 23 or 24 or 25 [2. study designs which would capture and may report data ] (4083402) | 2. study designs which would capture and may report data (even where not identified as natural history)  We focus on studies which track data over time, as indicated in guidance produced by the FDA.[121] These are represented in Lines 6 to 25.  We also include:   - ITS designs as these studies may track cohorts over time with the interruptions indicating data collection. - Cohort studies, where people known to have the condition or to support people with the condition have been tracked.   We search for incidence and prevalence. Whilst not strictly interested in these data for this review, the numbers returned by conclusion of this these search lines are low and it is possible that the studies in reporting data also capture data over time. |
| 27 (Osteogenesis imperfecta and year*).ti. (69) 28 ((200* adj3 201*) or (200* adj3 202*) or (201* adj3 202*)).ab. (547965) 29 Disease progression/ or Progression.ti,ab,kf. (822816) 30 ("follow* up*" or "follow* on*" or "growth plane*" or "patient journey" or "clinical journey" or "over time" or "time course" or "life course" or "life span" or "natural course" or "end to* end" or "from* diagnosis" or "long term").ti,ab,kf. (2513777) 31 27 or 28 or 29 or 30 [3. terms for data collection over a time period] (3577457) | 3. terms for data collection over a time period  Line 27 focuses on the primary condition term and it will pick up any study which includes year in the title (e.g.[122-124]).  Line seeks to identify cohorts tracked over time, such as.[23] |
| 32 5 or 26 or 31 (6658156) 33 (2004* or 2005* or 2006* or 2007* or 2008* or 2009* or 2010* or 2011* or 2012* or 2013* or 2014* or 2015* or 2016* or 2017* or 2018* or 2019* or 2020* or 2021* or 2022* or 2023* or 2024*).dt,dp,ed,ep,yr. (22827954) 34 1 and 32 and 33 (1217) | Line 32 combines the three sets of terms for NH.  Line 33 reports the date limit 2004-2014.  Line 37 completes the search by combining:   - Terms for condition (Line 1) - Terms for NH (Line 32) - Date limit (Line 33) |

Search strings used to search Embase (via Ovid), on 24 March 2024 (date limit 1974 to 22.03.2024)

| **#** | **Searches** | **Results** |
| --- | --- | --- |
| 1 | *osteogenesis imperfecta/ or (Osteogenesis imperfecta or (brittle adj3 bones)).ti,ab,kf. | 7460 |
| 2 | Natural histor*.ti,ab,kf. | 81225 |
| 3 | [Osteogenesis Imperfecta/hi or History.ti.] | 0 |
| 4 | ((patient* or client or after or provid* or family or families) adj3 histor*).ti,ab,kf. | 299918 |
| 5 | 2 or 3 or 4 [1. terms for natural history or historical data] | 375995 |
| 6 | *retrospective study/ | 40780 |
| 7 | (retrospective* adj2 (stud* or review* or analysis or analysed)).ti,ab,kf. | 1220304 |
| 8 | chart review*.ti,ab,kf. or *"medical record review"/ | 119837 |
| 9 | (Medical record* or patient chart*1 or patient record* or registry or registries or claims data* or patient survey*).ti,ab,kf. or *register/ or *health survey/ | 679743 |
| 10 | *Prospective Studies/ | 39690 |
| 11 | (prospective* adj2 (stud* or review* or analysis or analysed)).ti,ab,kf. | 661480 |
| 12 | *case control study/ | 9113 |
| 13 | case control*.ti,ab,kf. | 221043 |
| 14 | *longitudinal study/ | 9446 |
| 15 | (longitudinal or longitudinally).ti,ab,kf. | 499908 |
| 16 | *cross-sectional study/ | 14905 |
| 17 | (cross section or cross sectionally).ti,ab,kf. | 39003 |
| 18 | *epidemiology/ | 48539 |
| 19 | time series.ti,ab,kf. | 55341 |
| 20 | *cohort analysis/ | 49510 |
| 21 | cohort*.ti,ab,kf. | 1560730 |
| 22 | Osteogenesis Imperfecta/ep [Epidemiology] | 99 |
| 23 | (epidemiology or epidemiological).ti,ab,kf. | 592197 |
| 24 | *incidence/ or Incidence.ti,ab,kf. | 1384535 |
| 25 | *Prevalence/ or Prevalence.ti,ab,kf. | 1190155 |
| 26 | 6 or 7 or 8 or 9 or 10 or 11 or 12 or 13 or 14 or 15 or 16 or 17 or 18 or 19 or 20 or 21 or 22 or 23 or 24 or 25 [2. study designs which would capture and may report data ] | 6016642 |
| 27 | (Osteogenesis imperfecta and year*).ti. | 85 |
| 28 | ((200* adj3 201*) or (200* adj3 202*) or (201* adj3 202*)).ab. | 980278 |
| 29 | Disease progression/ or Progression.ti,ab,kf. | 1229737 |
| 30 | ("follow* up*" or "follow* on*" or "growth plane*" or "patient journey" or "clinical journey" or "over time" or "time course" or "life course" or "life span" or "natural course" or "end to* end" or "from* diagnosis" or "long term").ti,ab,kf. | 3686685 |
| 31 | 27 or 28 or 29 or 30 [3. terms for data collection over a time period] | 5303876 |
| 32 | 5 or 26 or 31 | 9500502 |
| 33 | (2004* or 2005* or 2006* or 2007* or 2008* or 2009* or 2010* or 2011* or 2012* or 2013* or 2014* or 2015* or 2016* or 2017* or 2018* or 2019* or 2020* or 2021* or 2022* or 2023* or 2024*).yr. | 27454137 |
| 34 | 1 and 32 and 33 | 1878 |
| 35 | (conference abstract* or conference review or conference paper or conference proceeding).db,pt,su. | 5876713 |
| 36 | 34 not 35 | 1191 |

Search strings used to search Embase (conference search, via Ovid), on 24 March 2024 (date limit 1974 to 22.03.2024)

| # | Searches | Results |
| --- | --- | --- |
| 1 | *osteogenesis imperfecta/ or (Osteogenesis imperfecta or (brittle adj3 bones)).ti,ab,kf. | 7460 |
| 2 | Natural histor*.ti,ab,kf. | 81225 |
| 3 | [Osteogenesis Imperfecta/hi or History.ti.] | 0 |
| 4 | ((patient* or client or after or provid* or family or families) adj3 histor*).ti,ab,kf. | 299918 |
| 5 | 2 or 3 or 4 [1. terms for natural history or historical data] | 375995 |
| 6 | *retrospective study/ | 40780 |
| 7 | (retrospective* adj2 (stud* or review* or analysis or analysed)).ti,ab,kf. | 1220304 |
| 8 | chart review*.ti,ab,kf. or *"medical record review"/ | 119837 |
| 9 | (Medical record* or patient chart*1 or patient record* or registry or registries or claims data* or patient survey*).ti,ab,kf. or *register/ or *health survey/ | 679743 |
| 10 | *Prospective Studies/ | 39690 |
| 11 | (prospective* adj2 (stud* or review* or analysis or analysed)).ti,ab,kf. | 661480 |
| 12 | *case control study/ | 9113 |
| 13 | case control*.ti,ab,kf. | 221043 |
| 14 | *longitudinal study/ | 9446 |
| 15 | (longitudinal or longitudinally).ti,ab,kf. | 499908 |
| 16 | *cross-sectional study/ | 14905 |
| 17 | (cross section or cross sectionally).ti,ab,kf. | 39003 |
| 18 | *epidemiology/ | 48539 |
| 19 | time series.ti,ab,kf. | 55341 |
| 20 | *cohort analysis/ | 49510 |
| 21 | cohort*.ti,ab,kf. | 1560730 |
| 22 | Osteogenesis Imperfecta/ep [Epidemiology] | 99 |
| 23 | (epidemiology or epidemiological).ti,ab,kf. | 592197 |
| 24 | *incidence/ or Incidence.ti,ab,kf. | 1384535 |
| 25 | *Prevalence/ or Prevalence.ti,ab,kf. | 1190155 |
| 26 | 6 or 7 or 8 or 9 or 10 or 11 or 12 or 13 or 14 or 15 or 16 or 17 or 18 or 19 or 20 or 21 or 22 or 23 or 24 or 25 [2. study designs which would capture and may report data ] | 6016642 |
| 27 | (Osteogenesis imperfecta and year*).ti. | 85 |
| 28 | ((200* adj3 201*) or (200* adj3 202*) or (201* adj3 202*)).ab. | 980278 |
| 29 | Disease progression/ or Progression.ti,ab,kf. | 1229737 |
| 30 | ("follow* up*" or "follow* on*" or "growth plane*" or "patient journey" or "clinical journey" or "over time" or "time course" or "life course" or "life span" or "natural course" or "end to* end" or "from* diagnosis" or "long term").ti,ab,kf. | 3686685 |
| 31 | 27 or 28 or 29 or 30 [3. terms for data collection over a time period] | 5303876 |
| 32 | 5 or 26 or 31 | 9500502 |
| 33 | (2022* or 2023* or 2024*).yr. | 4203828 |
| 34 | 1 and 32 and 33 | 332 |
| 35 | (conference abstract* or conference review or conference paper or conference proceeding).db,pt,su. | 5876713 |
| 36 | 34 and 35 | 113 |
