# Supplementary material for: The natural history of osteogenesis imperfecta: a systematic review
Source: Bone Rep. 2026 Jun 5;29:101927. doi: 10.1016/j.bonr.2026.101927 (PMC13266223; doi:10.1016/j.bonr.2026.101927)
Supplement: Appendix A.2 — Study quality assessment [file mmc2.docx]

Appendix A.2. Study quality assessment

**Risk of bias of included case series (n=22) using a bespoke assessment method based on the Joanna Briggs Institute Checklist**

| Author | Were there clear criteria for inclusion in the case series? | Was the condition measured in a standard, reliable way for all participants included in the case series? | Were valid methods used for identification of the condition for all participants included in the case series? | Did the case series have consecutive inclusion of participants? | Did the case series have complete inclusion of participants? | Was there clear reporting of the demographics of the participants in this study? | Was there clear reporting of clinical information of the participants? | Were the outcomes or follow up results of cases clearly reported? | Was there clear reporting of the presenting site(s)/clinic(s) demographic information? | Was statistical analysis appropriate? |
| --- | --- | --- | --- | --- | --- | --- | --- | --- | --- | --- |
| Ahn et al. (2019) [65] | Y | Y | Y | Unclear | Unclear | Y | Y | Y | Y | Y |
| Anissipour et al. (2014) [51] | Y | Y | Y | Y | N (incomplete medical histories) | Y | Y | Y | Y | Y |
| Arponen et al. (2012) [56] | Y | Y | Y | Y | Y | Y | Y | Y | Y | Y |
| Aslan et al. (2016) [32] | Y | Y | Y | Y | Y | Y | Y | Y | Y | Y |
| Binh et al. (2017) [48] | Y | Y | Y | Y | Y | Y | Y | Y | Y | Y |
| Chen et al. (2023) [54] | Y | Y | Y | Y | N (incomplete radiograph histories) | Y | Y | Y | Y | Y |
| Darba et al. (2020) [55] | Y | Y | Y | Y | Y | Y | N | N | Y | Y |
| Gimeno-Martos et al. (2017) ^a^ [89] | Y | Y | Y | Y | Y | N | Unclear | Unclear | Y | Unclear |
| Graff et al. (2017) [70] | Y | Y | Y | Y | N (excluded due to surgeries) | Y | N | Y | Y | Y |
| Greeley et al. (2013) [39] | Y | Y | Y | Y | Y | Y | Y | Y | Y | Y |
| Hadef et al. (2023) [36] | Y | Y | Y | Y | Y | N | N | Y | Y | Y |
| Johnson et al. (2008) [37] | Y | Unclear (diagnostic method not explicitly reported) | Y | Y | Y | Y | N | N | Y | Y |
| Joshi et al. (2023) [23] | Y | Y | Y | Y | Y | N | Y | Y | Y | Y |
| Kok et al. (2013) [61] | Y | Y | Y | Y | Y | Y | Y | Y | Y | Y |
| Koumakis et al. (2022) [41] | Y | Y | Y | Y | N (incomplete medical records) | Y | Y | Y | Y | Y |
| Caudevilla Lafuente et al. (2020) [38] | Y | N | Y | Unclear | Unclear | Y | Y | Y | Y | Y |
| Martens et al. (2018) [84] | Y | Y | Y | N/A (cohort followed up from a previous study) | N (excluded for surgery, or dropouts) | Y | Y | Y | Y | Y |
| Obafemi et al. (2008) [125] | Y | Y | Y | Y | Y | Y | Y | Y | Y | Y |
| Paterson et al. (2006) [126] | Y | Y | Y | Y | Y | N | Y | Y | Y | Y |
| Pillion et al. (2008) [85] | Y | Y | Y | Y | Y | N | Y | Y | Y | Y |
| Semler et al. (2010) [58] | Y | N (cut-off measurement influences precision) | Y | N | N | Y | Y | Y | Y | Y |
| Sepulveda et al. (2017) [43] | Y | Unclear (diagnostic method not explicitly reported) | Y | N | Y | Y | Y | Y | Y | Y |
| Wei et al. (2022) [44] | Y | Y | Y | N (patient dropouts during study period) | N | Y | Y | Y | Y | Y |
| ^a^ Study was in Portuguese and had an English translation of the abstract with relevant data. As per our protocol, only English text could be extracted, therefore it is unclear whether some aspects of the quality assessment were met. | | | | | | | | | | |

Risk of bias of included cohort studies (n=22) using a bespoke assessment method based on the CASP Cohort Checklist

| Study | Did the study address a clearly focused research question? | Was the cohort recruited in an acceptable way? | Was the exposure measured to minimise bias? | Was the outcome accurately measured to minimise bias? | Have the authors identified all important confounding factors? | Have the authors taken account of the confounding factors in the design and/or analysis? | Was the follow up of subjects complete enough? | Was the follow up of subjects long enough? | What are the results of this study? | How precise are the results? |
| --- | --- | --- | --- | --- | --- | --- | --- | --- | --- | --- |
| Al-Agha et al. (2016) [40] | Y | Y | Y | N | N | N | Y | Y | Cyclic zoledronate infusions reduced fracture frequency and pain frequency, and improved quality of life and mobility in children and adolescents | Precise |
| Andersen et al. (2022) [66] | Y | Y | Y | Y | Y | Y | Y | Y | OA is more prevalent in OI than in the general population | Very precise |
| Arponen et al. (2015) [59] | Y | Y | Y | Y | Y | Y | Y | Not reported | Cranial base pathology prevalence is not affected by treatments for OI | Precise |
| Bains et al. (2019) [50] | Y | Y | N | Y | N | N | Y | N | BP treatment affects several health outcomes | Precise |
| Barber et al. (2019) [71] | Y | Y | y | Y | N | Y | Y | N | Growth curves in types III and IV | Precise |
| Cheung et al. (2011) [127] | Y | Y | Y | Y | N | Y | Y | Y | Evolution of cranial base anomalies in a cohort | Precise |
| Chhabra et al. (2023) [128] | Y | Y | Y | Y | Y | Y | Y | Y | Characterisation of hand and wrist fractures | Precise |
| Corio et al. (2023) ^a^ [33] | Y | Y | Y | Unclear | Y | Y | Unclear | Y | People with OI have a shorter life expectancy than the general population | Precise |
| Escobar et al. (2013)^b^ [34] | Y | N/A | Unclear | Unclear | Unclear | Unclear | Unclear | Unclear | Description of patient characteristics in a single centre | Not precise (from abstract) |
| Folkestad et al. (2018) [35] | Y | Y | Y | Y | Y | Y | Y | Y | Mortality rates Denmark | Precise |
| Folkestad et al. (2017) [49] | Y | Y | Unclear | Unclear | Y | Y | Y | Y | Fracture rates Denmark | Precise |
| Folkestad et al. (2016) [22] | Y | Y | Y | Y | Y | Y | Y | Y | Cardiovascular disease Denmark | Precise |
| Lykking et al. (2022) [129] | Y | Y | Y | Y | Y | Y | Y | Y | No increase in fracture risk during/post-partum compared to before birth | Not precise |
| Lyster et al. (2022) [81] | Y | Y | Y | Y | Y | Y | Y | Y | Eye problems compared to reference population | Precise |
| Martens et al. (2018) [84] | Y | Y | Y | Y | N | N | Y | Y | Longitudinal prospective assessment of hearing loss | Not precise (small sample, missing data) |
| Nicol et al. (2021) [68] | Y | Y | Y | Y | Y | N | Y | Not reported | Relationship between growth and CXM levels | Precise |
| Ozturk et al. (2022) [60] | Y | Y | Y | Y | Y | N | Y | Y | Growth in children with OI | Precise (some inconsistencies in data collection are noted) |
| Pinheiro et al. (2019) [130] | Y | Y | Y | Y | Y | Y | Y | Y | About cyclic pamidronate treatment, but we focused on fracture patterns | Precise |
| Schramm et al. (2009) [42] | Y | Y | Y | Y | Y | Y | Y | y | In utero diagnosis of people with OI | Precise |
| Wei et al. (2022) [44] | Y | Y | Y | Y | Y | Y | Y | Y | Genotype-phenotype relationship in OI | Precise |
| Wilsford et al. (2013) [131] | Y | Y | Y | Y | Y | Y | Y | Y | Risk factors for vitamin D deficiency in children with OI | Precise |
| Xi et al. (2021) [123] | Y | Y | Y | Y | Y | Y | Y | Y | Genotype/phenotype characteristics of fractures in OI | Precise |
| Abbreviations: BP, bisphosphonates; CXM, C-terminal cross-linked telopeptide of type I collagen; OA, osteoarthritis; OI, osteogenesis imperfecta.  ^a^ Only the abstract was available and had relevant data, leading to its inclusion. Based only on the abstract, it is unclear whether certain aspects of the quality assessment were met.  b Study was in Portuguese and had an English translation of the abstract with relevant data. As per our protocol, only English text could be extracted, therefore it is unclear whether some aspects of the quality assessment were met. | | | | | | | | | | |

Risk of bias of included cross-sectional studies (n=23) using a bespoke assessment method based on the Joanna Briggs Institute Cross-Sectional Checklist

| Study | Were the criteria for inclusion in the sample clearly defined? | Were the study subjects and the setting described in detail? | Was the outcome measured in a valid and reliable way? | Were objective, standard criteria used for measurement of the condition? | Were confounding factors identified? | Were strategies to deal with confounding factors stated? | Were the outcomes measured in a valid and reliable way? | Was appropriate statistical analysis used? |
| --- | --- | --- | --- | --- | --- | --- | --- | --- |
| Bobak et al. (2023) [64] | Y | Y | Y | Y | Y | Y | Y | Y |
| Brizola et al. (2014) [47] | Y | Y | Y | Y | Y | Y | Y | Y |
| Da Costa et al. (2020) [82] | Y | Y | Y | Y | Y | Y | Y | Y |
| De Wouters et al. (2022) [46] | Y | Y | Y | Y | Y | y | y | y |
| Edouard et al. (2011) [62] | Y | Y | Y | Y | Y | Y | Y | Y |
| Edouard et al. (2011) [63] | Y | Y | Y | Y | Y | Y | Y | Y |
| Germain-Lee et al. 2016) [72] | Y | Y | Y | Y | N | N | Y | Y |
| Hald et al. (2018) [132] | Y | Y | Y | Y | Y | Y | Y | Y |
| Ltaief-Boudrigu et al. (2022) [83] | Y | N | Y | Y | Y | N | Y | Y |
| Machol et al. (2020) [74] | Y | Y | Y | Y | Y | Y | Y | Y |
| Mei et al. (2024) [67] | Y | Y | Y | Y | Y | Y | Y | Y |
| Radunovic et al. (2015) [76] | Y | Y | Y | Y | Y | Y | Y | Y |
| Rao et al. (2021) [80] | Y | Y | N (Self-reported) | N (self-reported) | N | N | N (self-reported) | Y |
| Sulko et al. (2005) a [133] | Unclear (not specified in abstract) | Y | Unclear (not specified in abstract) | Unclear (not specified in abstract) | Unknown | Unknown | Unclear (not specified in abstract) | Unknown |
| Swinnen et al. (2012) [88] | Y | Y | Y | Y | Y | Y | Y | Y |
| Swinnen et al. (2011) [86] | Y | Y | Y | Y | Y | Y | Y | Y |
| Tam et al. 2018) [77] | Y | Y | Y | Y | Y | N | Y | Y |
| Vuorimies et al. (2017) [134] | Y | N | Y | Y | N | N | Y | Y |
| Waissbluth et al. (2020) [87] | Y | Y | Y | Y | N | N | Y | Y |
| Watanbe et al. (2007) [53] | Y | N | Y | Y | Y | N | Y | Y |
| Yimgang et al. (2015) [69] | Y | Y | N (self-reported) | N (self-reported) | Y | N | Y | Y |
| Rodriguez Celin et al. (2023) [73] | Y | Y | Y | Y | Y | Y | Y | Y |
| Yimgang (2016) [79] | Y | N | N | N | Y | N | N | Y |
| ^a^ Only the abstract was available and had relevant data, leading to its inclusion. Based only on the abstract, it is unclear whether certain aspects of the quality assessment were met. | | | | | | | | |
