# Supplementary material for: The natural history of osteogenesis imperfecta: a systematic review
Source: Bone Rep. 2026 Jun 5;29:101927. doi: 10.1016/j.bonr.2026.101927 (PMC13266223; doi:10.1016/j.bonr.2026.101927)
Supplement: Appendix A.3 — Detailed summary of included studies with their characteristics. [file mmc3.docx]

Appendix A.3. Detailed summary of included studies with their characteristics.

Summary characteristics of studies included in this review

| **Author** | **Country** | **Study period** | **Centre** | **Data type** | **Retrospective** | **Total population size, n** | **Age** | **OI type/severity** |
| --- | --- | --- | --- | --- | --- | --- | --- | --- |
| Ahn et al. (2019) [65] | United States | 5.6 years (average) | Single centre specialised | Longitudinal | Retrospective | 55 | All ages:  Mean, 18 | I,10  III, 17  IV, 18  V, 2  Other, 5  Unknown, 3 |
| Al-Agha et al. (2016) [40] | Kingdom of Saudi Arabia | 12 years | Single centre specialised | Longitudinal | Retrospective | 72 | Range: 0–20  Mean: 9.65 | NR |
| Andersen et al. (2022) [66] | Denmark | 42 years | Registry - National | Longitudinal | Retrospective | 907 | All ages | NR |
| Anissipour et al. (2014) [51] | United States | 30 years | Single centre general | Longitudinal | Retrospective | 157 | All ages (scoliosis outcomes only for children) | I, 62  III, 55  IV, 32  Other, 8 |
| Arponen et al. (2012) [56] | Finland | 20.5 years mean follow-up | Single centre general | Longitudinal and cross-sectional | Retrospective | 76 | All ages:  Range, 0–39 | I, 47  III, 13  IV, 16 |
| Arponen et al. (2015) [59] | Finland | NR | Single centre specialised | Longitudinal | Retrospective | 39 | All ages:  Range, 0–24.8 | Mild, 20  Moderate, 10  Severe, 9 |
| Aslan et al. (2016) [32] | Turkey | NR | Single centre general | Longitudinal | Retrospective | 28 | All ages:  Median, 7.48 | I, 13  III, 10  IV, 5 |
| Bains et al. (2019) [50] | United States | 5 years | Registry - National | Longitudinal | Retrospective | 466 | All ages | I, 219  III, 79  IV 139  V, 13  Other, 16 |
| Barber et al. (2019) [71] | United States | 20 years | Single centre specialised | Longitudinal | Retrospective | 100 | Children | III, 45  IV, 55 |
| Binh et al. (2017) [48] | Vietnam | NR | Registry - National | Longitudinal and cross-sectional | Retrospective and prospective | 146 | All ages | I, 46  III, 46  IV, 54 |
| Bobak et al. (2023) [64] | United States | Not specified | Registry - National | Longitudinal | Retrospective | 8444 | All ages | NR |
| Brizola et al. (2014) [47] | Brazil | 9 months | Single centre specialised | Cross-sectional | Prospective | 62 | Children | I, 31  III, 9  IV, 22 |
| Caudevilla Lafuente et al. (2020) [38] | Spain | NR | Single centre general | Longitudinal | Retrospective | 40 | All ages | Mild, 27  Moderate, 8  Severe, 5 |
| Chen et al. (2023) [54] | Hong Kong | 8 years | Single centre general | Longitudinal | Retrospective | 290 | All ages | I, 39  III, 67  IV, 162  V, 22 |
| Cheung et al. (2011) [127] | Finland | 6.8 years (median) | Single centre specialised | Longitudinal and cross-sectional | Retrospective | 187 | All ages:  Median, 12  Range, 3.4–47 | I, 88  III, 30  IV, 51  V, 11  Other, 7 |
| Chhabra et al. (2023) [128] | United States | 6 years 2 months | Single centre general | Longitudinal | Retrospective | 469 | Children:  Mean, 10.44  SD, 4.95 | NR |
| Corio et al. (2023) [33] | Taiwan | 11 years | Registry - National | Longitudinal | Retrospective | 319 | All ages | NR |
| da Costa Otavio et al. (2020) [82] | Brazil | NR | Single centre specialised | Cross-sectional | Prospective | 77 | All ages:  Mean, 21.9  SD, 14.3  Range, 5–55 | I, 57  III, 5  IV, 13  Unknown, 2 |
| Darba et al. (2020) [55] | Spain | 18 years | Multi-centre | Longitudinal | Retrospective | 998 | All ages:  Mean, 13.19 | NR |
| De Wouters et al. (2022) [46] | Belgium | NR | Single centre general | Cross-sectional | Prospective | 22 | All ages:  Mean, 11  SD, 4.8 | I, 13  III, 3  IV, 5  V, 1 |
| Edouard et al. (2011) [63] | Canada | NR | Single centre general | Cross-sectional | Retrospective | 71 | All ages | I, 29  III, 12  IV, 30 |
| Edouard et al. (2011) [62] | Majority Canada | NR | Single centre specialised | Cross-sectional | Retrospective | 315 | Children | I, 165  III, 56  IV, 94 |
| Escobar et al. (2013) [34] | Portugal | NR | Single centre general | Longitudinal | Retrospective | 21 | NR | All |
| Folkestad et al. (2018) [35] | Denmark | 37 years | Registry - National | Longitudinal | Retrospective | 687 | All ages | NR |
| Folkestad et al. (2017) [49] | Denmark | 17.9 years median observation time | Registry - National | Longitudinal | Retrospective | 644 | All ages:  Median, 32.6  IQR, 15.3–51.3 | NR |
| Folkestad et al. (2016) [22] | Denmark | 37 years | Registry - National | Longitudinal | Retrospective | 687 | All ages | NR |
| Germain-Lee et al. (2016) [72] | United States | NR | Single centre specialised | Longitudinal and cross-sectional | Retrospective | 343 | All ages | I, 223  III, 70  IV, 36  V, 14 |
| Gimeno-Martos et al. (2017) [89] | Spain | 17 years | Registry - National | Longitudinal | Retrospective | 145 | All ages | NR |
| Graff et al. (2017) [70] | Poland | 6 years | Single centre general | Longitudinal | Retrospective | 117 | Children | NR |
| Greeley et al. (2013) [39] | United States | NR | Single centre specialised | Longitudinal | Retrospective | 68 | Children | I, 23  IV, 17  IV, 24 |
| Hadef et al. (2023) [36] | Algeria | 6 years | Single centre general | Cross-sectional | Prospective | 12 | All ages | NR |
| Hald et al. (2018) [132] | Denmark | N/A | Single centre general | Cross-sectional | Retrospective and prospective | 85 | Adults:  Mean, 45  Range, 19–78 | I, 58  III, 12  IV, 15 |
| Johnson et al. (2008) [37] | Oman | 8 years | Single centre general | Longitudinal | Retrospective | 22 | Children | NR |
| Joshi et al. (2023) [23] | Australia | 12 years | Single centre general | Longitudinal | Retrospective | 44 | Children:  Median, 11.3  IQR, 6.2–17 | I, 44 |
| Kok et al. (2013) [61] | Netherlands | 9 years (mean) | Single centre specialised | Longitudinal and cross-sectional | Retrospective | 74 | Children | I, 44  III/IV, 30 |
| Koumakis et al. (2022) [41] | France | 13 years 11 months | Multi-centre | Longitudinal | Retrospective | 50 | Adults | I, 78%  III, 13%  IV, 5%  V, 3% |
| Ltaief-Boudrigua et al. (2022) [83] | France | NR | Single centre specialised | Cross-sectional | Prospective | 41 | Adults:  Mean, 40  SD, 12  Range, 23–79 | NR |
| Lykking et al. (2022) [129] | Denmark | 24 years | Registry - National | Longitudinal | Retrospective | 111 | Adults:  Median, 28^a^  IQR, 25–31^a^ | NR |
| Lyster et al. (2022) [81] | Denmark | 44 years | Registry - National | Longitudinal | Retrospective | 907 | NR | NR |
| Machol et al. (2020) [74] | United States | NR | Multi-centre | Cross-sectional | Prospective | 491 | All ages:  Mean, 19  SD, 14.6  Range, 3.1–67.3 | I, 219  III, 87  IV, 141  V, 17  Other, 14  Unknown, 13 |
| Martens et al. (2018) [84] | Belgium | 4 years 1 month (average) | Single centre specialised | Longitudinal | Prospective | 31 | Adults:  Mean, 38.2  SD, 14 | I, 24  III, 3  IV, 4 |
| Mei et al. (2024) [67] | China | NR | Single centre specialised | Cross-sectional | Prospective | 23 | Adults:  Mean, 39.54  SD, 12.18 | I, 20  III, 0  IV, 2  V, 1 |
| Nicol et al. (2021) [68] | United States | NR | Multi-centre | Longitudinal | Prospective | 44 | Children | I, 44 |
| Obafemi et al. (2008) [125] | United States | 4–20 years of data per patient | Multi-centre | Longitudinal | Retrospective | 45 | Children | III, 25  IV, 20 |
| Ozturk et al. (2022) [60] | Turkey | NR | Single centre general | Longitudinal | Retrospective | 83 | Children | I, 43  III, 36  IV, 3  V, 1 |
| Paterson et al. (2006) [126] | UK | 10 years | Court proceedings | Longitudinal | Retrospective | 12 | Children (<18 years) | NR |
| Pillion et al. (2008) [85] | United States | 10 years | Single centre general | Longitudinal | Prospective | 41 | All ages:  Mean, 26.54  SD, 19.55  Range, 2.67–68 | NR^b^ |
| Pinheiro et al. (2019) [130] | Brazil | 10 years | Single centre specialised | Longitudinal | Retrospective | 45 | Children | All |
| Radunovic et al. (2015) [76] | Norway | NR | Registry - National | Cross-sectional | Retrospective | 99 | Adults:  Mean, 43.9  SD, 12.3 | I, 77  III, 10  IV, 11 |
| Rao et al. (2021) [80] | United States | NR | Registry - National | Survey | Prospective | 132 | Adults:  Median, 42.5 | Mild, 74.4%  Moderate, 24%  Severe, 1.6% |
| Rodriguez Celin et al. (2023) [73] | United States | NR | Multi-centre | Longitudinal and cross-sectional | Prospective | 861 | All ages:  Mean, 27.16  Median, 13.87  Range, 0.09–81.63 | I, 409  III, 164  IV, 223  Other, 65 |
| Schramm et al. (2009) [42] | Germany / Switzerland | 22 years | Single centre general | Longitudinal | Prospective | 35 | Neonates | III/IV, 178 |
| Semler et al. (2010) [58] | Canada | NR | Single centre specialised | Longitudinal and cross-sectional | Retrospective | 195 | All ages:  Median, 12  Range, 0.4–47.5 | I, 89  III, 27  IV, 50  V, 15  Other, 14 |
| Sepulveda et al. (2017) [43] | Chile | NR | Single centre general | Longitudinal | Retrospective | 67 | Children | I, 67 |
| Sułko et al. (2005) [133] | Poland | NR | NR | Longitudinal | Prospective | 141 | Children | NR |
| Swinnen et al. (2011) [86] | Netherlands | 2 years (time of cross-sectional data collection) | Single centre specialised | Cross-sectional | Retrospective | 184 | All ages:  Mean, 30.5  SD, 16.9  Range, 3–89 | I, 154  III, 4  IV, 26 |
| Swinnen et al. (2012) [88] | Belgium | NR | Single centre specialised | Cross-sectional | Retrospective | 182 | All ages:  Mean, 30.2  SD, 16.9  Range, 3–89 | I, 152  III, 4  IV, 26 |
| Tam et al. (2018) [77] | United States | NR | Multi-centre | Cross-sectional | Prospective | 209 | All ages:  Mean, 27.2  Range, 6.1–67.2 | I, 104  III, 37  IV, 52  V, 16 |
| Vuorimies et al. (2017) [134] | Finland | NR | Single centre general | Cross-sectional | Prospective | 50 | Children | NR |
| Waissbluth et al. (2020) [87] | Chile | NR | Single centre general | Cross-sectional | Prospective | 30 | All ages:  Mean, 22  SD, 12.7  Range, 6–63 | I, 20  III, 6  IV, 4 |
| Watanabe et al. (2007) [53] | Japan | NR | Single centre specialised | Cross-sectional | Retrospective | 19 | Children:  Mean, 14.2  Range, 4–20 | I, 7  III, 12 |
| Wei et al. (2022) [44] | China | 6 years | Single centre general | Longitudinal | Prospective | 116 | All ages | I, 14  III, 24  IV, 58  V, 5  Other, 15 |
| Wilsford et al. (2013) [131] | United States | 4 years 3 months | Single centre specialised | Longitudinal | Retrospective | 44 | Children | I, 15  III, 12  IV, 17 |
| Xi et al. (2021) [123] | China | 5 years (average) | Single centre general | Longitudinal | Retrospective and prospective | 19 | All ages:  Mean, 16.55  Median, 17  Range, 1–64 | I, 11  III, 3  IV, 5 |
| Yimgang et al. (2015) [69] | United States | NR | Registry - National | Cross-sectional | Retrospective | 274 | Adults | I, 216  III, 15  IV, 39  V, 2 |
| Yimgang et al. (2016) [79] | International | NR | Registry - National | Survey | Prospective | 77 | Neonates | I, 60  III, 4  IV, 13 |
| Abbreviations: OI, osteogenesis imperfecta.  Notes: ^a^ Age at first delivery. ^b^ Data was not extractable. | | | | | | | | |
