# Supplementary material for: The natural history of osteogenesis imperfecta: a systematic review
Source: Bone Rep. 2026 Jun 5;29:101927. doi: 10.1016/j.bonr.2026.101927 (PMC13266223; doi:10.1016/j.bonr.2026.101927)
Supplement: Appendix A.4 — Total number of fractures in OI cohort over 17.9-year observation period, by sex [file mmc4.docx]

Appendix A.4. Total number of fractures in OI cohort over 17.9-year observation period, by sex

Total number of fractures in OI cohort (n=644) over 17.9-year observation period by sex

Notes: Adapted from Folkestad et al., 2017. [49].
