# Supplementary material for: The natural history of osteogenesis imperfecta: a systematic review
Source: Bone Rep. 2026 Jun 5;29:101927. doi: 10.1016/j.bonr.2026.101927 (PMC13266223; doi:10.1016/j.bonr.2026.101927)
Supplement: Appendix A.5 — Median BMD Z-score in cohort of 83 children with OI [file mmc5.docx]

Appendix A.5. Median BMD Z-score in cohort of 83 children with OI

Median BMD Z-score in cohort of 83 children with OI

Notes: Adapted from Ozturk et al., 2022. [60]. In the cohort of children with OI Type III, four children were not present for a second evaluation, denoted by the two cohort sizes on the x-axis.
