# Supplementary material for: The natural history of osteogenesis imperfecta: a systematic review
Source: Bone Rep. 2026 Jun 5;29:101927. doi: 10.1016/j.bonr.2026.101927 (PMC13266223; doi:10.1016/j.bonr.2026.101927)
Supplement: Appendix A.6 — Median height Z-score in children and adults by OI type [file mmc6.docx]

Appendix A.6. Median height Z-score in children and adults by OI type

**Median height Z-score in children and adults by OI type**

Notes: Adapted from Germain-Lee et al., 2016. [72].
