# Supplementary material for: The natural history of osteogenesis imperfecta: a systematic review
Source: Bone Rep. 2026 Jun 5;29:101927. doi: 10.1016/j.bonr.2026.101927 (PMC13266223; doi:10.1016/j.bonr.2026.101927)
Supplement: Appendix A.7 — Age and gait onset by OI type [file mmc7.docx]

Appendix A.7. Age and gait onset by OI type

Age and gait onset by OI type

Notes: Adapted from Brizola et al., 2014. [47].
